# Gestural and symbolic development among apes and humans: support for a multimodal theory of language evolution

**DOI:** 10.3389/fpsyg.2014.01228

**Published:** 2014-10-30

**Authors:** Kristen Gillespie-Lynch, Patricia M. Greenfield, Heidi Lyn, Sue Savage-Rumbaugh

**Affiliations:** ^1^Department of Psychology, College of Staten Island, City University of New YorkNew York, NY, USA; ^2^Department of Psychology, University of California, Los AngelesLos Angeles, CA, USA; ^3^Department of Psychology, University of Southern MississippiLong Beach, MS, USA; ^4^President of Bonobo HopeDes Moines, IA, USA

**Keywords:** gestural theory of language evolution, language-enculturated apes, symbolic development, cross-species comparisons, gesture, communication development, language development, multimodal theory of language evolution

## Abstract

What are the implications of similarities and differences in the gestural and symbolic development of apes and humans?This focused review uses as a starting point our recent study that provided evidence that gesture supported the symbolic development of a chimpanzee, a bonobo, and a human child reared in language-enriched environments at comparable stages of communicative development. These three species constitute a complete clade, species possessing a common immediate ancestor. Communicative behaviors observed among all species in a clade are likely to have been present in the common ancestor. Similarities in the form and function of many gestures produced by the chimpanzee, bonobo, and human child suggest that shared non-verbal skills may underlie shared symbolic capacities. Indeed, an ontogenetic sequence from gesture to symbol was present across the clade but more pronounced in child than ape. Multimodal expressions of communicative intent (e.g., vocalization plus persistence or eye-contact) were normative for the child, but less common for the apes. These findings suggest that increasing multimodal expression of communicative intent may have supported the emergence of language among the ancestors of humans. Therefore, this focused review includes new studies, since our 2013 article, that support a multimodal theory of language evolution.

Darwin (1871) viewed fire and language as the greatest discoveries of humankind. Coincidentally, the first recorded attempt to teach apes language occurred when Garner (1900) taught a juvenile chimpanzee, Moses, to approximately vocalize “feu” (fire in French). Subsequent research has revealed that bonobos, chimpanzees, gorillas and orangutans are capable of communicating linguistically (despite limitations on vocabulary and syntactic complexity) when raised in language-enriched environments (Patterson, 1978; Savage-Rumbaugh et al., 1986; Miles, 1990). This research has contributed to the recognition that language is multi-faceted and that many (and possibly all) aspects of language are not uniquely human (Fitch et al., 2005).

In a recent paper, we provided evidence that gesture supports the symbolic development of apes and humans reared in language-enriched environments (Gillespie-Lynch et al., 2013). **Symbolic communication** is often defined as the intentional use of conventionalized tokens (such as words or visual symbols) to refer to, or stand in for, classes of entities or events that are distinct from the tokens (Savage-Rumbaugh et al., 1986; Deacon, 1998). Unlike **indexical communication** (wherein the meaning of a gesture is dependent on its context, such as pointing) and **iconic communication** (wherein the form or motion of an action or object is depicted, such as pantomime), symbols have meaning that is not as dependent on the context in which they occur. Our study was the first to use the same methods to compare the gestures of humans and apes at comparable stages in development. This review highlights research about human and ape gestural and symbolic development that motivated our study, describes our findings, integrates new research published since our 2013 article, and concludes with the implications of our own work and that of others for the gestural and multimodal theories of the evolution of language. New findings confirm the importance of gesture in the evolution of human language, but they also strengthen our conclusion that language evolution was a **multimodal process**.

KEY CONCEPT 1. Symbolic communication Symbolic communication is often defined as the intentional use of conventionalized tokens to refer to classes of entities or events.

KEY CONCEPT 2. Indexical communication Indexical gestures are gestures that depend on their context to give them meaning, such as pointing

KEY CONCEPT 3. Iconic communication Iconic gestures are gestures that depict the form or motion of an action or object, such as pantomimes.

KEY CONCEPT 4. Multimodal process Multimodal communication is the integrated use of signals from more than one modality, such as a sound paired with a visual gesture.

## The evolution of language

Because communicative behaviors such as language and gesture do not fossilize, theories about the evolution of language are inherently speculative. Communicative capacities observed across members of a clade (sibling species with a common ancestor, such as humans, chimpanzees, and bonobos) are likely inherited from a common ancestor while skills only apparent in one species, especially when they are raised in similar environments, are unlikely to have been present in the common ancestor (Parker and McKinney, 1999). The *presence* of a capacity across the clade suggests that the capacity existed in a common ancestor. Differences in the *frequency* of a behavior among humans relative to other apes, given similar rearing conditions, suggest that the capacity was enhanced as humans diverged from their sibling species.

**Cladistic analysis** suggests that neural structures underlying language may be shared across the clade. Despite millions of years of distinct evolutionary pressures, all species of great apes exhibit population level brain lateralization in perisylvian regions important for language learning (Cantalupo et al., 2003), a trait that does not seem to be shared with several monkey species, at least for the planum temporale (Lyn et al., 2011b).

KEY CONCEPT 5. Cladistic analysis Cladistic analysis is a method used to identify characteristics that were likely and unlikely to have been present in a common ancestor by comparing members of a clade, or species descended from that common ancestor.

A challenge facing researchers investigating the evolution of language is shifting definitions of language itself. Language is often defined circularly as whatever aspects of communication are uniquely human. While psychologists and biologists often define language as culturally specific shared systems of meaning, linguists often define language in terms of innate cognitive structures. A cross-disciplinary dialogue aimed at bridging these definitional chasms set forth two domains of language: a faculty of language in the broad sense (FLB) that consists of skills shared with other species and a faculty of language in the narrow sense (FLN) that is specific to humans (Fitch et al., 2005). They concluded that what constitutes FLN (including whether or not any aspects of language are unique to humans) and how language evolved remain open questions.

## The gestural theory of the evolution of language

The idea that language evolved from a primarily gestural mode of communication is centuries old (Condillac, 1746; Hewes, 1973; Corballis, 2002). Evidence for a gestural origin of language includes the relatively early emergence of bipedalism (freeing up the hands to gesture), the possibility that modern hand configurations arose much earlier than the modern vocal tract, the variability and flexibility of non-human primates' gestures relative to their vocal communication, shared neural substrates for manual action and language, and enhanced laterality of communicative gestures relative to other types of action (Greenfield, 1991, 2008; Lieberman, 1998; Rizzolatti and Arbib, 1998; Corballis, 2002; Hopkins et al., 2005; Molnar-Szakacs et al., 2006; Armstrong and Wilcox, 2007). Despite their flexibility, ape gestures often have specific meanings (Hobaiter and Byrne, 2014). Gestures produced by captive orangutans and captive and wild gorillas and chimpanzees are often used intentionally to serve consistent functions, as indexed by persistence on the part of the gesturer and the recipient's response (Genty et al., 2009; Cartmill and Byrne, 2010; Roberts et al., 2012a).

Although non-human primates use and respond to calls referring to specific aspects of the environment, these calls may be limited in their referential flexibility and the degree to which they are under voluntary control (Cheney and Seyfarth, 2010). Non-human primates may have a limited capacity for vocal imitation relative to humans, aquatic mammals and birds (Fitch, 2000). Indeed, early attempts to teach apes verbal speech revealed pronounced constraints in their capacity to say words. In the early 1900s, Furness (1916) attempted to teach two juvenile chimpanzees and orangutans to speak. After extensive training, one of the orangutans learned to say “cup” and clung to him while she said “papa.” Over the next 60 years, attempts to teach apes speech by co-rearing them with humans resulted in productive vocabularies of only a few utterances despite years of training (Kellogg and Kellogg, 1933; Hayes, 1951; Laidler, 1980). Despite the apes' great difficulty uttering words, researchers believed that the apes' comprehension of language far exceeded their ability to produce it.

In 1966, the Gardner and Gardner (1969) adopted a wild-captured female, 10 month-old chimpanzee, Washoe. They speculated that difficulties teaching apes to speak might be attributable to physiological rather than cognitive limitations. Relative to other apes, the human larynx is lower and the tongue is more flexible; this allows humans to produce a wider range of discriminable sounds than other apes (Lieberman, 1968; Fitch, 2000). Washoe's caregivers used a simple form of American Sign Language (ASL) to sign about daily activities. Like human children, Washoe learned language by engaging in shared routines with caregivers (Gardner and Gardner, 1969; Acredelo and Goodwyn, 1988). Although caregivers taught her to imitate (or copy) gestures, immediate imitation was more effective for shaping signs than for introducing novel signs. While some signs were acquired via delayed imitation, ontogenetic ritualization (or shaping of previously uncommunicative behaviors into communicative signals through repeat interactions between individuals) probably played a greater role in her symbolic development than imitation. Indeed, Washoe's early signs were often shaped from spontaneous behaviors, such as pounding on the door to request that it be opened.

As Washoe's productive vocabulary increased to approximately 150 signs by 8 years, more names, pragmatic functions and meaningful word combinations emerged. When introduced into a colony of chimpanzees who were encouraged to sign together, Washoe employed techniques that humans had used to teach signs to her—molding, modeling, and signing on his body—to teach signs to Loulis, an infant chimpanzee who she adopted (Fouts and Fouts, 1989). This contrasts with reports that wild juvenile apes learn by observing adults who do not directly teach them (Matsuzawa, 2002).

Subsequent research demonstrated that additional chimpanzees, gorillas, and orangutans could acquire substantial productive vocabularies when gestural or lexigram (arbitrary visual symbols) rather than oral communication systems were used (Hayes, 1951; Gardner and Gardner, 1969; Patterson, 1978; Savage-Rumbaugh, 1987; Patterson et al., 1988; Fouts and Fouts, 1989; Miles, 1990; Bonvillian and Patterson, 1993). These apes grouped symbols into categories when their referent was absent, combined symbols into meaningful novel statements, exhibited word order preferences, and demonstrated the range of pragmatic functions exhibited by children including using language to deceive, to indicate novelty, to communicate declaratively, and to refer to non-present entities (Greenfield and Smith, 1976; Patterson, 1978; Gardner and Gardner, 1969; Greenfield and Savage-Rumbaugh, 1984, 1990; Savage-Rumbaugh, 1987; Miles, 1990; Lyn, 2007; Lyn et al., 2011a,c). While it is important to note that one can group symbols into categories based on perceptual similarity without an understanding of their symbolic functions (e.g., Vygotsky, 1962), errors during vocabulary tests provided additional evidence that language-enculturated apes represent lexigrams (which typically bear no perceptual similarity to their referent) in terms of semantic (but not syntactic) categories (Gardner and Gardner, 1969; Lyn, 2007).

Thus, ape language research provided strong support for the gestural theory of language by demonstrating that apes could communicate symbolically with visual rather than auditory communication systems. However, human language may involve integration of information across different modalities (Hickok and Poeppel, 2007). Indeed, recent evidence suggests that multimodal communication, or the ability to integrate information across different modalities, may have supported the evolution of language (Taglialatela et al., 2011).

## A call for a multimodal theory of the evolution of language

Although evidence suggests that non-human primates use gestures more flexibly than sounds (Pollick and de Waal, 2007), this evidence may be distorted by a tendency to focus on monkeys in the wild when assessing vocalizations and on apes in captivity when assessing gestures (Slocombe et al., 2011). Recent findings suggest that chimpanzee alarm calls are intentional: they direct them more frequently to allies, persist until their allies are out of danger, and monitor responses (Schel et al., 2013). Both monkeys and apes are more likely to call around certain individuals, suggesting volitional control of vocalizations (Arbib et al., 2008). In addition, non-human primates' comprehension of vocal cues may be substantially more flexible than production (Seyfarth and Cheney, 2010). Apes exhibit referential flexibility by using and/or responding to sequences of calls as if they convey more information than the individual calls of which they are composed (Clarke et al., 2006; Clay and Zuberbühler, 2011). Although teaching apes to produce human sounds, which their vocal tracts are not designed to do, was ineffective, teaching apes to modify their own vocalizations into a set of sounds that a computer can discern as associated with different referents has not been systematically attempted but is theoretically possible.

While previous research suggested that chimpanzee gestures activate neural regions associated with language, this association was found only among the apes in the study who vocalized while gesturing (Taglialatela et al., 2011). Recent research has shown that chimpanzees in captivity may learn attention-getting calls socially and can be operantly conditioned to use calls that were not previously in their repertoire (Taglialatela et al., 2012; Russell et al., 2013). These findings have prompted calls for a multimodal theory of language evolution wherein language may have evolved from an integrated system of vocal, facial, and gestural signals (Slocombe et al., 2011).

Foundational to a multimodal theory of language acquisition is the neural integration of hands and mouth. In 1991, Greenfield proposed just such an integration. She noted that, in the first two years of human life, a common neural substrate (roughly Broca's area) underlies the organization of elements in both speech and manual action. The theory posited that an evolutionary homolog of the neural substrate for language production and manual action has provided a foundation for the evolution of human language. Support for this proposition comes from the discovery of a Broca's area homolog and related neural circuits in contemporary primates. More recent research has revealed an even tighter connection: semantic information conveyed by speech and gesture is processed in the same brain areas (reviewed in Levinson and Holler, 2014).

Although the study that is the focus of this review was designed to evaluate the gestural theory of language evolution, our findings revealed unexpected support for the multimodal theory of language evolution. In retrospect, a multimodal theory of language evolution is more logical than a purely gestural theory because the human brain is essentially a multi-modal device that converts different modalities of input (e.g., light, sound, touch) into an interpretable framework in order to respond to it. Primates and many other animals integrate information across multiple sensory modalities (Pack and Herman, 1995; Wallace et al., 1996). Given that the input that primates respond to is multimodal, it stands to reason that they would produce multimodal output. Therefore, the *capacity* for multimodal communication was present in the common ancestor of apes and humans. It seems only logical that any language-based system that evolved would make use of the input and output modalities of the biological systems already in place and honed by millions of years of evolution. A key and unexpected finding of the study that is the focus of this review is that the *frequency* of multimodal communication may be greater in humans relative to apes when they are raised in similar rearing conditions. This suggests that multimodal communication was enhanced as humans diverged from their sibling species and that this enhancement may have supported the evolution of language.

## A developmental investigation of the gestural theory of language evolution

The study that is the focus of this review was designed to evaluate the gestural theory of language evolution by examining if gesture supports symbolic development across the clade. While phylogeny (evolution) does not repeat ontogeny (development), later stages of development cannot evolve without the ontogenetic foundation of earlier stages already being present; and, thus, later stages of ontogenetic development also tend to evolve later (Parker and McKinney, 1999). Thus, a longitudinal comparison of gestural and symbolic development across the clade provides essential information about the role of gesture in language evolution.

### Evidence that gesture supports language development and language evolution

Gesture is a precursor to symbolic communication for both humans and language-enculturated apes (Brakke and Savage-Rumbaugh, 1996; Iverson and Goldin-Meadow, 2005). Deictic gestures (context-dependent indication) help infants understand links between symbols and referents, allow infants to refer to objects before mastering their names, are more common than words early in development, and predict linguistic development in typical and atypical humans across cultures (Bates et al., 1975; Caselli and Volterra, 1990; Goldin-Meadow and Morford, 1994; Iverson and Goldin-Meadow, 2005; Rowe et al., 2008; Colonnesi et al., 2010; Iverson, 2010; Özçalışkan and Goldin-Meadow, 2010; Goldin-Meadow and Alibali, 2013).

Representational gestures (referring to a specific referent irrespective of context) may also predict linguistic development (Acredolo and Goodwyn, 1985). However, iconic gestures, or non-arbitrary representational gestures wherein the form or motion of an action or object is depicted, typically emerge approximately 6 months *after* first verbs (Özçalışkan et al., 2013).

Infants initially use both gestures and words referentially, but the use of words to represent and gestures to indicate increases with development (Capirci and Volterra, 2008). While words typically become more common than gestures within the second year of life, gestures remain important as part of two-element combinations (Greenfield and Smith, 1976; Iverson et al., 1994; Capirci and Volterra, 2008). Infants refer to objects in the gestural modality before they refer to them with speech and gesture-symbol combinations precede the development of symbol-symbol combinations in human children and language-enculturated apes (Greenfield and Smith, 1976; Iverson and Goldin-Meadow, 2005; Greenfield et al., 2008).

A primary aim of our study was to determine if this pattern of gestures preceding words was apparent across the clade. A secondary aim was to compare the gestures of humans and apes at comparable stages of development, as no previous study had used video data to compare the gestures of a bonobo, chimpanzee, and human at comparable stages of communicative development.

### Similarities and differences between ape and human gestures: a developmental perspective

Unlike the gestures of human children, the majority of ape gestures are dyadic, intended to draw another's attention to oneself, rather than triadic, intended to draw another's attention to an external entity (Pika, 2008). Also unlike most humans, ape gestures are frequently imperative (requests) and less frequently declarative (attempts to share experience with another; Lyn et al., 2011a). Nonetheless, declarative deictic gestures have been observed throughout the clade: in humans (e.g., Greenfield and Smith, 1976); chimpanzees, both language-trained (Lyn et al., 2011a) and in the wild (Hobaiter et al., 2014); and bonobos, both language trained (Lyn et al., 2011a) and in the wild (Vea and Sabater-Pi, 1998; Leavens, 2004).

As is the case for some (but not all) of the iconic gestures of human children (Acredolo and Goodwyn, 1985), iconic gestures can emerge spontaneously in bonobos and gorillas. Savage-Rumbaugh et al. (1977) pioneered in describing spontaneous iconic gestures used by bonobos. Among a group of captive bonobos, they identified seven different iconic gestures used to position two partners for copulatory bouts, such as moving one's hand and forearm across one's body to induce one's partner to turn around. Gestural combinations were also used to induce a partner to turn around, e.g., first touching a part of the partner's body, then moving one's hand and forearm across one's own body. A gesture that is both iconic and indexical, the “directed scratch gesture,” has also been observed among captive bonobos (Pika and Mitani, 2006), while a beckoning gesture combining iconic and indexical (or deictic) elements has been described for bonobos in a Congo sanctuary (Genty and Zuberbuhler, 2014). Iconic gestures have also been reported among captive and language-enculturated gorillas (Liebal and Pika, 2005; Tanner et al., 2006).

Another similarity between apes and humans is the ability chimpanzees, bonobos and 1-year-old children have to use gestures to communicate about absent and displaced objects (Liszkowski et al., 2009; Lyn et al., 2014; Roberts et al., 2014). Because of flawed methodology, displaced reference was not observed among apes in earlier research (Liszkowski et al., 2009). Thus, displaced reference, a hallmark of language, is visible across the clade in the gestural modality.

How gestures develop remains controversial. One thing that is clear is that social interaction is important for gestural development of both human infants (Acredelo and Goodwyn, 1988) and chimpanzees (Bard et al., 2014). While imitation, or social learning through observation, is believed to play a strong role in human gestural development (e.g., Caselli and Volterra, 1990), some human gestures may emerge independently of imitation (Acredelo and Goodwyn, 1988). Imitation may contribute far less to the gestural development of apes relative to humans, as variability in gestures is typically similar within and across groups of apes (Liebal and Call, 2012). Group-specific gestures have been observed, albeit infrequently, among captive and wild apes suggesting some gestural imitation (Pika and Liebal, 2006; Hobaiter and Byrne, 2011).

Ontogenetic ritualization, or conventionalization, may underlie much of ape gestural development (Halina et al., 2013), although this is not without controversy. This is a process of mutual anticipation in which particular social behaviors come to function as intentional communicative signals within a dyad. For example, Plooij (1979) documented how a mother's act of raising a baby chimpanzee's arm to groom him was subsequently transformed into a conventionalized gesture performed by the baby: he raised his arm to ask mother to groom him. This same type of conventionalization is described by Bruner (1975) for young children wherein a mother initiates an interactive routine and the infant signals for the recurrence of the routine by performing some portion of it. However, evidence suggests that ontogenetic ritualization contributes very little to human gestural development (Marentette and Nicoladis, 2012).

Although the specific forms they take may be culturally determined, the general form of many gestures may be inherited from the common ancestor of humans and apes. Researchers documented 66 distinct gestures over 266 days of observation of wild chimpanzees (Hobaiter and Byrne, 2011). Almost half of the gestures had been documented in other ape species. A study classifying the gestures of wild chimpanzees based on their form revealed similar gestures to those documented in other captive and wild ape populations, as well as among humans such as “arm beckoning” and “hand clapping” (Roberts et al., 2012b).

Despite the lack of prior research directly comparing the gestures of apes and humans, purported differences in the gestures of apes and humans have been used to support assertions of human uniqueness. For instance, (Seidenberg and Petitto, 1987) stated that apes do not produce iconic gestures, which may indicate that they cannot mentally represent entities, and that finger pointing requires an ability to draw attention to specific aspects of the environment that apes may lack. Similarly, Povinelli et al. (2003) asserted that apes do not *really* point because they lack an understanding of other minds. Tomasello (2007) also asserted that both declarative pointing and human language derive from a uniquely human capacity to understand others' minds and share experiences. He stated that apes differ from human children in that they do not share experience for its own sake, as evinced by the absence of showing gestures among apes.

However, substantial evidence contradicts the assertion that pointing is unique to humans. Index finger pointing has been observed among captive apes (Leavens and Hopkins, 1999), language-enculturated apes (Miles, 1990; Brakke and Savage-Rumbaugh, 1996; Krause and Fouts, 1997; Tanner et al., 2006) and apes in the wild (Inoue-Nakamura and Matsuzawa, 1997; Vea and Sabater-Pi, 1998). Early immersion in language-enriched environments may facilitate finger pointing among apes (Call and Tomasello, 1994). Finger pointing is infrequently observed among captive or wild apes who have not been language-enculturated, but reaching is commonly demonstrated by captive and wild apes (e.g., Leavens and Hopkins, 1999; Roberts et al., 2012b). Functional pointing without index finger extension has also been observed; chimpanzees in the wild use “directed scratches” to request grooming of specific body parts (Pika and Mitani, 2006). “Directed scratches” combine an indexical and an iconic element. Reaching and such “directed scratches” generally are used to request, whereas finger pointing often signifies indication. These findings suggest that ape gestures are more frequently imperative than the gestures of humans and that early exposure may increase declarative gesturing among apes, a point confirmed by other research (see below).

Similarly, it has been suggested that comprehension of declarative pointing is unique to humans among members of the clade consisting of bonobos, chimpanzees, and humans (Moll and Tomasello, 2007). This suggestion has been surprising, particularly given other species' (such as dogs and dolphins) ability to follow pointing gestures (e.g., Miklosi and Soproni, 2006; Pack and Herman, 2007). However, several studies have pointed to methodological differences as the largest driver of differences between apes and other animals in comprehension of declarative pointing (Mulcahy and Call, 2009; Lyn, 2010; Mulcahy and Hedge, 2012). Meta-analyses showed that when apes were tested with more distant object referents (as most other species had been) their comprehension of pointing was similar to that documented among other species (Mulcahy and Hedge, 2012). Comprehension of declarative gesturing also seems to be supported by language-enriched environments, with apes from language-enriched environments outperforming captive apes on distant and near pointing tasks (Lyn et al., 2010).

Although infrequently observed, showing gestures have also been reported for a language-enculturated gorilla (Bonvillian and Patterson, 1999). Showing, a clearly declarative gesture, precedes pointing for humans (Bates et al., 1975), but emerged after pointing for the gorilla, whose pointing behaviors were often interpreted as requests for a caregiver to perform an action. The reduced frequency of declarative gestures produced early in development by the gorilla relative to human children suggests that pointing may serve a more imperative function for apes than humans. Indeed, greater relative frequency of imperative vs. declarative gestures was previously documented among the participants in the study that is the focus of this review (e.g., Lyn et al., 2011a).

## Cladistic analysis of gestures: key findings and implications for language evolution

We adapted a study by Iverson and Goldin-Meadow (2005) to determine whether gestures support symbolic development across the clade consisting of bonobos, chimpanzees, and humans. Approximately an hour a month of video data of a language-enculturated bonobo (Panbanisha) and chimpanzee (Panpanzee) between 12 and 26 months of age was compared to videos of a human child (GN) between 11 and 18 months of age. Thus, approximately 14 and 15 h of video footage were coded for the bonobo and chimpanzee, respectively, compared to 8 h of video footage of the child.

Panbanisha and Panpanzee were raised together from soon after birth in a language-enriched environment wherein they were encouraged to communicate with lexigrams and gestures while engaging in mutually meaningful routines with caregivers (Brakke and Savage-Rumbaugh, 1996). Thus, the types of communicative input received by the apes and the human child were quite similar in that communication occurred during meaningful routines for all three species. However, input was not systematically controlled for and could have influenced our findings. Across 4 years, virtually all evidence of the apes' symbol production and comprehension was entered into a database at the end of the day. Actions preceded communicative gestures, primarily requests, for both apes. Gestures preceded communicative lexigram use for Panpanzee and co-emerged with communicative lexigram use for Panbanisha. Both apes exhibited comprehension and use of lexigrams across contexts indicating that lexigrams were not simply associative stimuli for them. Deferred imitation of prior dialogue provided a foundation for their emerging symbol combinations, as it did for two human children (Gillespie-Lynch et al., 2011). Combinations became increasingly independent of prior input among humans *and* apes with development.

In support of the gestural theory of language evolution, our study revealed pronounced similarities in the form and function of gestures produced by Panbanisha, Panpanzee, and the child (see Gillespie-Lynch et al., 2013). Increasing reliance on symbols relative to gestures was apparent with development, regardless of species. However, symbols became more frequent than gestures over the course of the study for the human but not the apes. Although all three species exhibited the predicted pattern of objects and events being more likely to be referred to via gesture before speech than the reverse, this pattern was only statistically significant for the human child, likely because she referred to more objects across modalities than did the apes.

However, findings also supported the multimodal theory of language evolution. Communicative intent, or evidence that a gesture was emitted or a symbol was used in order to influence another, is central to the definition of symbolic communication (e.g., Savage-Rumbaugh et al., 1986). All three species exhibited the same markers of communicative intent: eye gaze, vocalization, and persistence. Thus, these markers of communicative intent were likely present in our common ancestor. However, the child more frequently accompanied a gesture with more than one marker of communicative intent than the apes did. The species difference was particularly striking for multimodal communications consisting of gesture plus vocalization. Given that intentionality is a central aspect of symbolic communication, these differences in frequency suggest that increases in multimodal communication—particularly the combination of gesture plus vocalization—may have supported the evolution of language.

Findings also provided gestural evidence that ape communication is more instrumental than human communication. While all three species exhibited finger pointing, the human child pointed more and reached less (138 points vs. 151 reaches) than the bonobo (11 points vs. 271 reaches) and chimpanzee (17 points vs. 358 reaches). The human child was the only one to produce a number of gestures not exhibited by the other species including a declarative gesture, “show,” and an iconic gesture, “open.”

Similarities in types of gestures and the developmental progression from gesture to symbol across the clade when all three species were raised in language-enriched environments suggest that the common ancestor of chimpanzees, bonobos, and humans had the capacity to learn to use a range of gestures, including finger-pointing, and that gestures likely supported the evolution of language. Differences in the frequency of communicative signals and gestures in humans relative to the other apes suggest that the capacity to use multimodal communication (especially gesture plus vocalization), as well as declarative and iconic gestures, was enhanced as humans diverged from their sibling species. Given that the ancestors of humans initially developed language *without* access to a language-enriched environment, the increasing frequency of multimodal signals and specific gestures in the human line may have supported the emergence of language.

## Implications for the evolution of language: key roles for gesture and for multimodal communication

Our findings, in conjunction with prior research, demonstrate that gesture precedes symbolic development across the clade consisting of bonobos, chimpanzees, and humans. Our findings also suggest that a combination of shared ancestry (Hobaiter and Byrne, 2011; Roberts et al., 2012b), ontogenetic ritualization (Halina et al., 2013), and imitation (Gardner and Gardner, 1969; Caselli and Volterra, 1990; Miles, 1990; Pika and Liebal, 2006; Gillespie-Lynch et al., 2011) support communicative development across the clade. Differences in the frequency with which humans and apes use multimodal signals to indicate communicative intent, point, use iconic gestures, imitate others, and communicate declaratively suggest that increasing ability to engage in multimodal communication, especially gesture plus vocalization, may have scaffolded the evolution of language (Pika and Liebal, 2006; Lyn et al., 2011a; Gillespie-Lynch et al., 2013).

The child in our study paired gestures with multimodal indices of communicative intent, such as eye gaze, vocalization, and persistence, much more frequently than the apes. A pattern of apes using multimodal communication rarely is evident in research with captive and language-enculturated apes (Savage-Rumbaugh, 1987; Bodamar and Gardner, 2002; Leavens et al., 2010). For example, captive apes typically move into another's visual field before gesturing rather than capturing their attention through vocalization or touch (Liebal et al., 2004). However, wild chimpanzees vocalize while gesturing (Roberts et al., 2012b). Ape vocal, gestural and facial signals have typically been studied separately with different coding systems (Slocombe et al., 2011). Our study was one of the few to integrate coding across modalities, thus being able to assess the frequency of multimodal communication (Gillespie-Lynch et al., 2013).

Multimodal signals may elicit a stronger response than the sum of their parts (Slocombe et al., 2011). In infant development, abstraction is facilitated by multimodal cues, intersensory redundancy supports learning of arbitrary relations between vowels and objects, and multimodal information helps infants imitate speech, learn words (Legerstee, 1990; Gogate and Bahrick, 1998; Frank et al., 2009; Rader and Zukow-Goldring, 2010), and generate visual expectancies (Greenfield, 1972). Indeed, human language may be multimodal. McNeill (1992) asserts that speech-synchronized gestures should be considered part of speech.

Because the conclusion of our study was that language has a multimodal (particularly gestural + vocal) origin in both phylogeny and ontogeny, it is relevant to note how the visual and vocal modalities are integrated early in human language acquisition: Between one and two years of age, a common type of verbal communication is an indicative in which the child points at an entity and says its name (Greenfield and Smith, 1976). In similar fashion, bonobos in a sanctuary in the Republic of Congo coordinate sound and gesture into a unified communication. In such communications, sound and gesture complement each other to make up a complex meaning that is communicated to one or more conspecifics (Genty et al., 2014).

Although our findings suggest that gesture supported the evolution of language, they do not support the theory that iconic gestures supported the evolutionary transition from action through gesture to language (Tanner et al., 2006). Although traditional accounts of symbolic development suggest that iconicity supports word learning (e.g., Werner and Kaplan, 1984), iconic gestures emerge developmentally after verbs, perhaps because they require complex representational skills in order to decouple an action schema from an action goal and reinterpret it as something else (Özçalışkan et al., 2013). Acredolo and Goodwyn (1985) interpreted most of a child's early representational gestures as indexical (having shifting meanings depending on context) rather than iconic because they referred to routines wherein the gesture was learned. Many ape gestures that have been classified as iconic would be defined as indexical according to this definition. In infancy, Kanzi produced gestures that were interpreted as iconic (i.e., reach). These gestures likely indexed shared routines. Also contrary to the theory that language evolved from iconic gestural depictions of actions (e.g., Tanner et al., 2006), the early vocabularies of language-enculturated apes often include at least as many names of objects as references to actions (e.g., Brakke and Savage-Rumbaugh, 1996). Our findings and those of others suggest that indexical gestures (such as pointing) paired with multimodal signals of communicative intent are more likely candidates for the types of gestures that may have supported the evolution of language than iconic gestures.

Increasing use of indexical gestures paired with multimodal indices of communicative intent by the ancestors of humans may have produced more frequent and more compelling opportunities for the ancestors of humans to engage in shared attention toward external entities and objects. Engagement in shared attention is associated with enhanced language development among human children (e.g., Tomasello and Farrar, 1986). By encouraging shared attention, indexical gestures may have supported interpersonal environments conducive to the emergence of more symbolic (less context bound) communication. Indeed, a system of self-reflective indexes may underly symbolic communication more generally. Terrence Deacon states that, “language is made possible by a vast network of inter-referring indices… [that] effectively “point” to one another (1998, p. 401).” Indexical gestures paired with multimodal cues might have elicited stronger responses than gestures paired with fewer indices of communicative intent (e.g., Slocombe et al., 2011) and been more effective in supporting the types of abstractions that are essential to developing symbolic communication (e.g., Legerstee, 1990; Gogate and Bahrick, 1998; Frank et al., 2009; Rader and Zukow-Goldring, 2010).

Over a century of ape language research has yielded strong evidence in support of the importance of gesture in language evolution and revealed that apes can learn many aspects of language previously thought to be uniquely human when reared in language-enriched environments from infancy. Indeed, it remains possible that other species possess a form of language that has not yet been discerned. Early interactive routines, and opportunities to share attention more generally, may be central to the symbolic development of apes, as they are for human children (Acredelo and Goodwyn, 1988). In contrast to the varied ways that apes immersed in language-enriched environments use symbols, less flexible use of symbols is observed when apes are trained to communicate using operant conditioning (Terrace et al., 1979; Miles, 1983; Savage-Rumbaugh, 1987; O'Sullivan and Yeager, 1989). Our recent study added to prior research by providing evidence supporting the importance of gesture in the ontogeny and phylogeny of language, while, at the same time suggesting that the gestural theory of language evolution should be expanded to include a multimodal foundation for the evolution of human language.

## Author note

We dedicate this manuscript to the memory of Panpanzee and Panbanisha, both of whom were taken from us too soon. We would like to thank the reviewers and editors of this paper for their careful attention to detail and constructive feedback.

### Conflict of interest statement

The authors declare that the research was conducted in the absence of any commercial or financial relationships that could be construed as a potential conflict of interest.

## References

[B1] Acredelo L. Goodwyn S. (1988). Symbolic gesturing in normal infants. Child Dev. 59, 450–466. 10.2307/1130324 2452052

[B2] Acredolo L. P. Goodwyn S. W. (1985). Symbolic gesturing in language development. Hum. Dev. 28, 40–49. 10.1159/000272934 2452052

[B3] Arbib M. A. Liebal K. Pika S. (2008). Primate vocalization, gesture, and the evolution of human language. Curr. Anthropol. 49, 1053–1076. 10.1086/593015 19391445

[B4] Armstrong D. F. Wilcox S. E. (2007). The Gestural Origin of Language. Oxford: Oxford University Press

[B5] Bard K. A. Dunbar S. Maguire-Herring V. Veira Y. Hayes K. G. McDonald K. (2014). Gestures and social-emotional communicative development in chimpanzee infants. Am. J. Primatol. 76, 14–29. 10.1002/ajp.22189 24038115

[B6] Bates E. Camaioni L. Volterra V. (1975). Performatives prior to speech. Merrill Palmer Q. 21, 205–226. 24038115

[B6a] Bodamar M. D. Gardner R. A. (2002). How cross-fostered chimpanzees (*Pan troglodytes*) initiate and maintain conversations. J. Compara. Psychol. 116, 12–26. 10.1037/0735-7036.116.1.12 11926680

[B7] Bonvillian J. D. Patterson F. G. (1993). Early sign language acquisition in children and gorillas: vocabulary content and sign iconicity. First Lang. 13, 315–338. 10.1177/014272379301303903

[B8] Bonvillian J. D. Patterson F. G. (1999). Early sign-language acquisition: comparisons between children and gorillas, in The Mentalities of Gorillas and Orangutans: Comparative Perspectives, eds Parker S. T. Mitchell R. W. Miles H. L. (Cambridge, MA: Cambridge University Press), 240–264

[B9] Brakke K. E. Savage-Rumbaugh E. S. (1996). The development of language skills in *Pan*-II. Production. Lang. Commun. 16, 361–380. 10.1016/S0271-5309(96)00018-3

[B10] Bruner J. S. (1975). The ontogenesis of speech acts. J. Child Lang. 2, 1–19. 10.1017/S0305000900000866

[B11] Call J. Tomasello M. (1994). Production and comprehension of referential pointing by orangutans *(Pongo pygmaeus)*. J. Comp. Psychol. 108, 307–317. 10.1037/0735-7036.108.4.307 7813191

[B12] Cantalupo C. Pilcher D. L. Hopkins W. D. (2003). Are planum temporale and sylvian fissure asymmetries directly related? a MRI study in great apes. Neuropsychologia 41, 1975–1981. 10.1016/S0028-3932(02)00288-9 14572530

[B13] Capirci O. Volterra V. (2008). Gesture and speech. The emergence and development of a strong and changing partnership. Gesture 8, 22–44. 10.1075/gest.8.1.04cap

[B14] Cartmill E. A. Byrne R. W. (2010). Semantics of primate gestures: intentional meanings of orangutan gestures. Anim. Cogn. 13, 793–804. 10.1007/s10071-010-0328-7 20563619

[B15] Caselli M. C. Volterra V. (1990). From communication to language in hearing and deaf children, in From Gesture to Language in Hearing and Deaf Children, eds Volterra V. Erting C. J. (Berlin; Heidelberg: Springer-Verlag), 263–277

[B16] Cheney D. L. Seyfarth R. M. (2010). Primate communication and human language: continuities and discontinuities, in Mind the Gap, eds Kappeler P. M. Silk J. B. (Berlin; Heidelberg: Springer), 283–298

[B17] Clarke E. Reichard U. H. Zuberbühler K. (2006). The syntax and meaning of wild gibbon songs. PLoS ONE 1:e73. 10.1371/journal.pone.0000073 17183705 PMC1762393

[B18] Clay Z. Zuberbühler K. (2011). Bonobos extract meaning from call sequences. PLoS ONE 6:e18786. 10.1371/journal.pone.0018786 21556149 PMC3083404

[B19] Colonnesi C. Stams G. J. J. Koster I. Noom M. J. (2010). The relation between pointing and language development: a meta-analysis. Dev. Rev. 30, 352–366. 10.1016/j.dr.2010.10.001

[B20] Condillac E. B. (1746). Essai sur l'origine des con- naissances humaines, ouvrage ou l'on r6duit a un seul principe tout ce concerne l'entendement. Oeuvres philosophiques de Condillac. Paris: Georges LeRoy (1947).

[B21] Corballis M. C. (2002). From Hand to Mouth: The Origins of Language. Princeton: Oxford: Princeton University Press

[B22] Darwin C. (1871). The Descent of Man. London: John Murray

[B23] Deacon T. W. (1998). The Symbolic Species: The Co-evolution of Language and the Brain. New York, NY: WW Norton and Company

[B24] Fitch W. (2000). The evolution of speech: a comparative review. Trends Cogn. Sci. 4, 258–267. 10.1016/S1364-6613(00)01494-7 10859570

[B25] Fitch W. T. Hauser M. D. Chomsky N. (2005). The evolution of the language faculty: clarifications and implications. Cognition 97, 179–210. 10.1016/j.cognition.2005.02.005 16112662

[B26] Fouts R. S. Fouts D. H. (1989). Loulis in conversation with the cross-fostered chimpanzees, in Teaching Sign Language to Chimpanzees, eds Gardner R. A. Gardner B. T. Van Cantfort T. E. (Albany, NY: State University of New York Press), 293–307

[B27] Frank M. C. Slemmer J. A. Marcus G. F. Johnson S. P. (2009). Information from multiple modalities helps 5−month−olds learn abstract rules. Dev. Sci. 12, 504–509. 10.1111/j.1467-7687.2008.00794.x 19635078 PMC2718773

[B28] Furness W. H. (1916). Observations on the mentality of chimpanzees and orang-utans. Proc. Am. Philos. Soc. 55, 281–290

[B29] Gardner R. A. Gardner B. T. (1969). Teaching sign language to a chimpanzee. Science 165, 664–672. 10.1126/science.165.3894.664 5793972

[B30] Garner R. L. (1900). Apes and Monkeys: Their Life and Language. Boston, MA; London, UK: Ginn & company

[B31] Genty E. Breuer T. Hobaiter C. Byrne R. W. (2009). Gestural communication of the gorilla (*Gorilla gorilla*): repertoire, intentionality and possible origins. Anim. Cogn. 12, 527–546. 10.1007/s10071-009-0213-4 19184669 PMC2757608

[B32] Genty E. Clay Z. Hobaiter C. Zuberbuhler K. (2014). Multi-modal use of a socially directed call in bonobos. PLoS ONE 9:e84738. 10.1371/journal.pone.0084738 24454745 PMC3893130

[B33] Genty E. Zuberbuhler K. (2014). Spatial reference in a bonobo gesture. Curr. Biol. 24, 1601–1605. 10.1016/j.cub.2014.05.065 24998531

[B34] Gillespie-Lynch K. Greenfield P. M. Feng Y. Savage-Rumbaugh S. Lyn H. (2013). A cross-species study of gesture and its role in symbolic development: implications for the gestural theory of language evolution. Front. Psychol. 4:160. 10.3389/fpsyg.2013.00160 23750140 PMC3674957

[B34a] Gillespie-Lynch K. Greenfield P. M. Lyn H. Savage-Rumbaugh S. (2011). The role of dialogue in the ontogeny and phylogeny of early symbol combinations: a cross-species comparison of bonobo, chimpanzee, and human learners. First Lang. 31, 442–460. 10.1177/0142723711406882

[B35] Gogate L. J. Bahrick L. E. (1998). Intersensory redundancy facilitates learning of arbitrary relations between vowel sounds and objects in seven-month-old infants. J. Exp. Child Psychol. 69, 133–149. 10.1006/jecp.1998.2438 9637756

[B36] Goldin-Meadow S. Alibali M. W. (2013). Gesture's role in speaking, learning, and creating language. Annu. Rev. Psychol. 64, 257. 10.1146/annurev-psych-113011-143802 22830562 PMC3642279

[B37] Goldin-Meadow S. Morford M. (1994). Gestures in early child language, in From Gesture to Language in Hearing and Deaf Children, eds Volterra V. Erting C. J. (Berlin; New York: Springer), 249–262

[B38] Greenfield P. M. (1972). Playing peek-a-boo with a four-month-old: a study of the role of speech and nonspeech sounds in the development of a visual schema. J. Psychol. 82, 287–298. 10.1080/00223980.1972.9923819 4655557

[B39] Greenfield P. M. (1991). Language, tools, and brain: the ontogeny and phylogeny of hierarchically organized sequential behavior. Behav. Brain Sci. 14, 531–551. 10.1017/S0140525X00071235

[B40] Greenfield P. M. (2008). Implications of mirror neurons for the ontogeny and phylogeny of cultural processes: the examples of tools and language, in Action to Language Via the Mirror Neuron System, ed Arbib M. (Cambridge: Cambridge University Press), 501–533

[B41] Greenfield P. M. Lyn H. Savage-Rumbaugh E. S. (2008). Protolanguage in ontogeny and phylogeny. Combining deixis and representation. Interact. Stud. 9, 34–50. 10.1075/is.9.1.04gre

[B42] Greenfield P. M. Savage-Rumbaugh E. S. (1984). Perceived variability and symbol use: a common language-cognition interface in children and chimpanzees. J. Comp. Psychol. 98, 201–218. 10.1037/0735-7036.98.2.201 6744815

[B43] Greenfield P. M. Savage-Rumbaugh E. S. (1990). Grammatical combination in *Pan paniscus*: processes of learning and invention in the evolution and development of language, in Comparative Developmental Psychology of Language and Intelligence in Primates, eds Parker S. Gibson K. (Cambridge: Cambridge University Press), 540–578

[B44] Greenfield P. M. Smith J. H. (1976). The Structure of Communication in Early Language Development. New York, NY: Academic Press

[B45] Halina M. Rossano F. Tomasello M. (2013). The ontogenetic ritualization of bonobo gestures. Anim. Cogn. 16, 653–666. 10.1007/s10071-013-0601-7 23370783

[B46] Hayes C. (1951). The Ape in Our House. New York, NY: Harper

[B47] Hewes G. W. (1973). Primate communication and the gestural origin of language. Curr. Anthropol. 14, 5–25. 10.1086/201401

[B48] Hickok G. Poeppel D. (2007). The cortical organization of speech processing. Nat. Rev. Neurosci. 8, 393–402. 10.1038/nrn2113 17431404

[B49] Hobaiter C. Byrne R. (2014). The meanings of chimpanzee gestures. Curr. Biol. 24, 1596–1600. 10.1016/j.cub.2014.05.066 24998524

[B50] Hobaiter C. Byrne R. W. (2011). The gestural repertoire of the wild chimpanzee. Anim. Cogn. 14, 745–767. 10.1007/s10071-011-0409-2 21533821

[B51] Hobaiter C. Leavens D. A. Byrne R. W. (2014). Deictic gesturing in wild chimpanzees (*Pan troglodytes*)? Some possible cases. J. Comp. Psychol. 128, 82–87. 10.1037/a0033757 24040760

[B52] Hopkins W. D. Russell J. Freeman H. Buehler N. Reynolds E. Schapiro S. J. (2005). The distribution and development of handedness for manual gestures in captive chimpanzees *(Pan troglodytes)*. Psychol. Sci. 16, 487–493. 10.1111/j.0956-7976.2005.01561.x 15943676 PMC2043162

[B53] Inoue-Nakamura N. Matsuzawa T. (1997). Development of stone tool use by wild chimpanzees *(Pan trodglodytes)*. J. Comp. Psychol. 111, 159–173. 10.1037/0735-7036.111.2.159 9170281

[B54] Iverson J. M. (2010). Developing language in a developing body: the relationship between motor development and language development. J. Child Lang. 37, 229–261. 10.1017/S0305000909990432 20096145 PMC2833284

[B55] Iverson J. M. Capirci O. Caselli M. C. (1994). From communication to language in two modalities. Cogn. Dev. 9, 23–43. 10.1016/0885-2014(94)90018-3

[B56] Iverson J. M. Goldin-Meadow S. (2005). Gesture paves the way for language development. Psychol. Sci. 16, 367–371. 10.1111/j.0956-7976.2005.01542.x 15869695

[B57] Kellogg W. N. Kellogg L. A. (1933). The Ape and the Child: A Study of Environmental Influence Upon Early Behavior. New York, NY; London, UK: Hafner Publishing Co

[B58] Krause M. A. Fouts R. S. (1997). Chimpanzee (*Pan troglodytes)* Pointing: hand shapes, accuracy, and the role of eye gaze. J. Comp. Psychol. 111, 330–336. 10.1037/0735-7036.111.4.330 9419879

[B59] Laidler K. (1980). The Talking Ape. London, UK: Collins

[B60] Leavens D. A. (2004). Manual deixis in apes and humans. Interact. Stud. 5, 387–408. 10.1075/is.5.3.05lea

[B61] Leavens D. A. Hopkins W. D. (1999). The whole-hand point: the structure and function of pointing from a comparative perspective. J. Comp. Psychol. 113, 417–425. 10.1037/0735-7036.113.4.417 10608565 PMC2080771

[B62] Leavens D. A. Russell J. L. Hopkins W. D. (2010). Multimodal communication by captive chimpanzees (Pan troglodytes). Anim. Cogn. 13, 33–40. 10.1007/s10071-009-0242-z 19504272 PMC2797826

[B63] Legerstee M. (1990). Infants use multimodal information to imitate speech sounds. Infant Behav. Dev. 13, 343–354. 10.1016/0163-6383(90)90039-B

[B64] Levinson S. C. Holler J. (2014). The origin of human multimodal communication. Philos. Trans. R. Soc. B Biol. Sci. 369, 1–9. 10.1098/rstb.2013.0302 PMC4123681 25092670

[B65] Liebal K. Call J. (2012). The origins of non-human primates' manual gestures. Philos. Trans. R. Soc. B Biol. Sci. 367, 118–128. 10.1098/rstb.2011.0044 22106431 PMC3223783

[B66] Liebal K. Call J. Tomasello M. Pika S. (2004). To move or not to move: how apes adjust to the attentional state of others. Interact. Stud. 5, 199–219. 10.1075/is.5.2.03lie

[B67] Liebal K. Pika S. (2005). Hands-on communication: use of gestures in apes and humans, in Proceedings of Interacting Bodies Conference (Lyons).

[B68] Lieberman D. E. (1998). Sphenoid shortening and the evolution of modern cranial shape. Nature 393, 158–162. 10.1038/30227 9603517

[B69] Lieberman P. (1968). Primate vocalizations and human linguistic ability. J. Acoust. Soc. Am. 44, 1574. 10.1121/1.1970384 5702032

[B69a] Liszkowski U. Schäfer M. Carpenter M. Tomasello M. (2009). Prelinguistic infants, but not chimpanzees, communicate about absent entities. Psychol. Sci. 20, 654–660. 10.1111/j.1467-9280.2009.02346.x 19476595

[B70] Lyn H. (2007). Mental representation of symbols as revealed by vocabulary errors in two bonobos (Pan paniscus). Anim. Cogn. 10, 461–475. 10.1007/s10071-007-0086-3 17436026

[B71] Lyn H. (2010). Environment, methodology, and the object choice task in apes: evidence for declarative comprehension and implications for the evolution of language. J. Evol. Psychol. 8, 333–349. 10.1556/JEP.8.2010.4.3

[B72] Lyn H. Greenfield P. M. Savage-Rumbaugh E. S. (2011c). Semiotic combinations in *Pan:* a comparison of communication in a chimpanzee and two bonobos. First Lang. 31, 300–325. 10.1177/0142723710391872 PMC3079886 21516208

[B73] Lyn H. Greenfield P. M. Savage-Rumbaugh S. Gillespie-Lynch K. Hopkins W. D. (2011a). Nonhuman primates do declare! A comparison of declarative symbol and gesture use in two children, two bonobos, and a chimpanzee. Lang. Commun. 31, 63–74. 10.1016/j.langcom.2010.11.001 21516208 PMC3079886

[B74] Lyn H. Pierre P. J. Bennett A. J. Fears S. C. Woods R. P. Hopkins W. D. (2011b). Planum temporale grey matter asymmetries in chimpanzees (Pan troglodytes), vervet (Chlorocebus aethiops sabaeus), rhesus (Macaca mulatta) and bonnet (Macaca radiata) monkeys. Neuropsychologia 49, 2004–2012. 10.1016/j.neuropsychologia.2011.03.030 21447349 PMC3151738

[B75] Lyn H. Russell J. L. Hopkins W. D. (2010). The impact of environment on the comprehension of declarative communication in apes. Psychol. Sci. 21, 360–365. 10.1177/0956797610362218 20424069 PMC6348075

[B76] Lyn H. Russell J. L. Leavens D. A. Bard K. A. Boysen S. T. Schaeffer J. A.. (2014). Apes communicate about absent and displaced objects: methodology matters. Anim. Cogn. 17, 85–94. 10.1007/s10071-013-0640-0 23681052 PMC3818454

[B77] Marentette P. Nicoladis E. (2012). Does ontogenetic ritualization explain early communicative gestures in human infants? in Developments in Primate Gesture Research, eds Pika S. Liebal K. (Amsterdam: John Benjamins Publishing), 33–53

[B78] Matsuzawa T. (2002). Chimpanzee Ai and her son Ayumu: an episode of education by master-apprenticeship, in The Cognitive Animal: Empirical and Theoretical Perspectives on Animal Cognition, eds Bekoff M. Allen C. Burghardt G. (Cambridge, MA: The MIT Press), 189–195

[B78a] McNeill D. (1992). Hand and Mind: What Gestures Reveal About Thought. Chicago, IL: University of Chicago Press

[B79] Miklosi A. Soproni K. (2006). A comparative analysis of animals' understanding of the human pointing gesture. Anim. Cogn. 9, 81–93. 10.1007/s10071-005-0008-1 16235075

[B80] Miles H. L. (1983). Apes and language: the search for communicative competence, in Language in Primates, eds de Luce J. Wilder H. T. (New York, NY: Springer), 43–61

[B81] Miles H. L. (1990). The cognitive foundations for reference in a signing orangutan, in “Language” and Intelligence in Monkeys and Apes: Comparative Developmental Perspectives, eds Parker S. T. Gibson K. R. (Cambridge: Cambridge University Press), 511–539

[B82] Moll H. Tomasello M. (2007). Cooperation and human cognition: the Vygotskian intelligence hypothesis. Philos. Trans. R. Soc. B Biol. Sci. 362, 639–648. 10.1098/rstb.2006.2000 17296598 PMC2346522

[B83] Molnar-Szakacs I. Kaplan J. Greenfield P. M. Iacaboni M. (2006). Observing complex action sequences: the role of the fronto-parietal mirror neuron system. Neuroimage 33, 923–935. 10.1016/j.neuroimage.2006.07.035 16997576

[B84] Mulcahy N. J. Call J. (2009). The performance of bonobos (Pan paniscus), chimpanzees (Pan troglodytes), and orangutans (Pongo pygmaeus) in two versions of an object-choice task. J. Comp. Psychol. 123, 304–309. 10.1037/a0016222 19685972

[B85] Mulcahy N. J. Hedge V. (2012). Are great apes tested with an abject object-choice task? Anim. Behav. 83, 313–321. 10.1016/j.anbehav.2011.11.019

[B86] O'Sullivan C. Yeager C. P. (1989). Communicative context and linguistic competence: the effect of social setting on a chimpanzee's conversational skill, in Teaching Sign Language to Chimpanzees, eds Gardner R. A. Gardner B. T. Van Cantfort T. E. (Albany, NY: SUNY Press), 269–279

[B87] Özçalşkan S. Gentner D. Goldin-Meadow S. (2013). Do iconic gestures pave the way for children's early verbs? Appl. Psycholinguist. 35, 1–20. 10.1017/S0142716413000325 25309008 PMC4188428

[B87a] Özçalşkan Ş. Goldin-Meadow S. (2010). Sex differences in language first appear in gesture. Dev. Sci. 13, 752–760. 10.1111/j.1467-7687.2009.00933.x 20712741 PMC2923747

[B88] Pack A. A. Herman L. (2007). The Dolphin's (Tursiops truncatus) understanding of human gazing and pointing: knowing what and where. J. Comp. Psychol. 121, 34–45. 10.1037/0735-7036.121.1.34 17324073

[B89] Pack A. A. Herman L. M. (1995). Sensory integration in the bottlenosed dolphin: immediate recognition of complex shapes across the senses of echolocation and vision. J. Acoust. Soc. Am. 98, 722–733. 10.1121/1.413566 7642811

[B90] Parker S. T. McKinney M. L. (1999). Origins of Intelligence: The Evolution of Cognitive Development in Monkeys, Apes, and Humans. Baltimore, MD: The Johns Hopkins University Press

[B91] Patterson F. Tanner J. Mayer N. (1988). Pragmatic analysis of gorilla utterances: early communicative development in the gorilla Koko. J. Pragmat. 12, 35–54. 10.1016/0378-2166(88)90018-5

[B92] Patterson F. G. (1978). The gestures of a gorilla: language acquisition in another pongid. Brain Lang. 5, 72–97. 10.1016/0093-934X(78)90008-1 618570

[B93] Pika S. (2008). Gestures of apes and pre-linguistic human children: Similar or different? First Lang. 28, 116–140. 10.1177/0142723707080966

[B94] Pika S. Liebal K. (2006). Differences and similarities between the natural gestural communication of the great apes and human children, in The Evolution of Language: Proceedings of the 6th International Conference (EVOLANG6) (New Jersey, NJ: World Scientific), 267–274

[B95] Pika S. Mitani J. (2006). Referential gestural communication in wild chimpanzees *(Pan troglodytes)*. Curr. Biol. 16, R191–R192. 10.1016/j.cub.2006.02.037 16546066

[B96] Plooij F. (1979). Some basic traits of language in wild chimpanzees? in Action, Gesture, and Symbol: The emergence of Language, ed Lock A. (London: Academic Press), 111–132

[B97] Pollick A. S. de Waal F. B. (2007). Ape gestures and language evolution. Proc. Natl. Acad. Sci. U.S.A. 104, 8184–8189. 10.1073/pnas.0702624104 17470779 PMC1876592

[B98] Povinelli D. Bering J. Giambrone S. (2003). Chimpanzee ‘pointing’: another error of the argument by analogy? in Pointing: Where Language, Culture, and Cognition Meet, ed Kita S. (Hillsdale, NJ: Erlbaum), 35–68

[B99] Rader N. D. V. Zukow-Goldring P. (2010). How the hands control attention during early word learning. Gesture 10, 202–221. 10.1075/gest.10.2-3.05rad

[B100] Rizzolatti G. Arbib M. (1998). Language within our grasp. Trends Neurosci. 21, 188–194. 10.1016/S0166-2236(98)01260-0 9610880

[B101] Roberts A. I. Vick S. J. Buchanan-Smith H. M. (2012a). Usage and comprehension of manual gestures in wild chimpanzees. Anim. Behav. 84, 459–470. 10.1016/j.anbehav.2012.05.022

[B102] Roberts A. I. Vick S. J. Roberts S. G. B. Buchanan-Smith H. M. Zuberbühler K. (2012b). A structure-based repertoire of manual gestures in wild chimpanzees: statistical analyses of a graded communication system. Evol. Hum. Behav. 33, 578–589. 10.1016/j.evolhumbehav.2012.05.006

[B103] Roberts A. I. Vick S. J. Roberts S. G. B. Menzel C. R. (2014). Chimpanzees modify intentional gestures to coordinate search for hidden food. Nat. Commun. 5, 3088. 10.1038/ncomms4088 24430433 PMC4350813

[B104] Rowe M. L. Özçalşkan Ş. Goldin-Meadow S. (2008). Learning words by hand: Gesture's role in predicting vocabulary development. First Lang. 28, 182–199. 10.1177/0142723707088310 19763249 PMC2745165

[B105] Russell J. McIntyre J. Hopkins W. Taglialatela J. (2013). Vocal learning of a communicative signal in captive chimpanzees, *Pan troglodytes*. Brain Lang. 127, 520–525. 10.1016/j.bandl.2013.09.009 24144730 PMC3982915

[B106] Savage-Rumbaugh E. S. (1987). Communication, symbolic communication, and language: reply to Seidenberg and Pettito. J. Exp. Psychol. Gen. 116, 288–292. 2442292 10.1037//0096-3445.116.3.288

[B107] Savage-Rumbaugh E. S. Wilkerson B. J. Bakeman R. (1977). Spontaneous gestural communication among conspecifics in the pygmy chimpanzee (*Pan paniscus*), in Progress in Ape Research, ed Bourne G. H. (New York, NY: Academic Press), 97–116

[B108] Savage-Rumbaugh S. McDonald K. Sevcik R. A. Hopkins W. D. Rubert E. (1986). Spontaneous symbol acquisition and communicative use by pygmy chimpanzees (Pan paniscus). J. Exp. Psychol. Gen. 115, 211. 10.1037/0096-3445.115.3.211 2428917

[B109] Schel A. M. Machanda Z. Townsend S. W. Zuberbühler K. Slocombe K. E. (2013). Chimpanzee food calls are directed at specific individuals. Anim. Behav. 86, 955–965. 10.1016/j.anbehav.2013.08.013

[B109a] Seidenberg M. S. Petitto L. A. (1987). Communication, symbolic communication, and language: comment on Savage-Rumbaugh, McDonald, Sevcik, Hopkins, and Rupert (1986). J. Exp. Psychol. Gen. 116, 279–287. 10.1037/0096-3445.116.3.279 2442292

[B110] Seyfarth R. M. Cheney D. L. (2010). Production, usage, and comprehension in animal vocalizations. Brain Lang. 115, 92–100. 10.1016/j.bandl.2009.10.003 19944456

[B111] Slocombe K. E. Waller B. M. Liebal K. (2011). The language void: the need for multimodality in primate communication research. Anim. Behav. 81, 919–924. 10.1016/j.anbehav.2011.02.002

[B112] Taglialatela J. Reamer L. Schapiro S. Hopkins W. (2012). Social learning of a communicative signal in captive chimpanzees. Biol. Lett. 8, 498–501. 10.1098/rsbl.2012.0113 22438489 PMC3391466

[B113] Taglialatela J. P. Russell J. L. Schaeffer J. A. Hopkins W. D. (2011). Chimpanzee vocal signaling points to a multimodal origin of human language. PLoS ONE 6:e18852. 10.1371/journal.pone.0018852 21533079 PMC3080370

[B114] Tanner J. E. Patterson F. G. Byrne R. W. (2006). The development of spontaneous gestures in zoo-living gorillas and sign-taught gorillas: from action and location to object representation. J. Dev. Process. 1, 69–103

[B115] Terrace H. S. Petitto L. A. Sanders R. J. Bever T. G. (1979). Can an ape create a sentence? Science 206, 891–902. 10.1126/science.504995 504995

[B116] Tomasello M. (2007). If they're so good at grammar, then why don't they talk? hints from apes' and humans' use of gestures. Lang. Learn. Dev. 3, 133–156. 10.1080/15475440701225451

[B117] Tomasello M. Farrar M. J. (1986). Joint attention and early language. Child Dev. 1454–1463. 10.2307/1130423 3802971

[B118] Vea J. J. Sabater-Pi J. (1998). Spontaneous pointing behavior in the wild pigmy chimpanzee *(Pan paniscus)*. Folia Primatol. 69, 289–290. 10.1159/000021640 9751833

[B119] Vygotsky L. S. (1962). Thought and Language, Edited and translated by Hanfmann E. Vakar G.. Cambridge, MA: MIT Press. 10.1037/11193-000

[B120] Wallace M. T. Wilkinson L. K. Stein B. E. (1996). Representation and integration of multiple sensory inputs in primate superior colliculus. J. Neurophysiol. 76, 1246–1266. 8871234 10.1152/jn.1996.76.2.1246

[B121] Werner H. Kaplan B. (1984). Symbol Formation. Hillsdale, NJ: Lawrence Erlbaum (First published in 1963).

